# Correlating nanoscale motion and ATP production in healthy and favism erythrocytes: a real-time nanomotion sensor study

**DOI:** 10.3389/fmicb.2023.1196764

**Published:** 2023-06-02

**Authors:** Marco Girasole, Simone Dinarelli, Giovanni Longo

**Affiliations:** Biotech@ISM, Istituto di Struttura della Materia, Consiglio Nazionale delle Ricerche, Rome, Italy

**Keywords:** erythrocytes, nanomotion sensor, favism, ATP - adenosine triphosphate, cellular metabolism, aging, real-time analysis

## Abstract

**Introduction:**

Red blood cells (RBCs) are among the simplest, yet physiologically relevant biological specimens, due to their peculiarities, such as their lack of nucleus and simplified metabolism. Indeed, erythrocytes can be seen as biochemical machines, capable of performing a limited number of metabolic pathways. Along the aging path, the cells’ characteristics change as they accumulate oxidative and non-oxidative damages, and their structural and functional properties degrade.

**Methods:**

In this work, we have studied RBCs and the activation of their ATP-producing metabolism using a real-time nanomotion sensor. This device allowed time-resolved analyses of the activation of this biochemical pathway, measuring the characteristics and the timing of the response at different points of their aging and the differences observed in favism erythrocytes in terms of the cellular reactivity and resilience to aging. Favism is a genetic defect of erythrocytes, which affects their ability to respond to oxidative stresses but that also determines differences in the metabolic and structural characteristic of the cells.

**Results:**

Our work shows that RBCs from favism patients exhibit a different response to the forced activation of the ATP synthesis compared to healthy cells. In particular, the favism cells, compared to healthy erythrocytes, show a greater resilience to the aging-related insults which was in good accord with the collected biochemical data on ATP consumption and reload.

**Conclusion:**

This surprisingly higher endurance against cell aging can be addressed to a special mechanism of metabolic regulation that permits lower energy consumption in environmental stress conditions.

## 1. Introduction

Red blood cells (RBCs) are among the simplest and most studied biological specimens. One of the reasons is that they lack a nucleus and synthetic apparatus, which make this system extremely intriguing under several points of view. Indeed, erythrocytes can basically be regarded as biochemical machines that perform a limited number of metabolic pathways required to ensure the accomplishment of their physiological role of O_2_/CO_2_ transporters. Due to their inability to self-replicate, the RBCs are assembled in the bone marrow and work in the bloodstream until the cellular damage accumulation impairs their structural integrity and their functionality, which, in turn, influence their interaction with the blood environment and determine the cell aging. In particular, during their life cycle, the cells change their morphological and biochemical properties, and the structural and functional properties of the cells degrade.

These cells have a few main peculiarities including an adapted metabolism that uses glucose instead of oxygen to synthetize ATP, and their unique shape-controlling structure called membrane-skeleton which provides the cells with the exceptional mechanical properties required for a proper physiological function. In their mature form, RBCs adopt a biconcave shape with a diameter of around 8 microns and a typical height of two microns. Their physiological role of oxygen transporter in the bloodstream makes them a crucial component for the health of a living organism (Girasole and Dinarelli, 2023).

The inability to modify, structurally or functionally, its proteome in response to different environmental conditions makes the RBC an interesting indicator of the cardiovascular condition of a person and the characteristics of its aging pathway are particularly meaningful in this view. Indeed, there is a growing consensus regarding the connection between the aging of an organism and the cellular aging, especially from the point of view of the cellular metabolism.

Previous works on the erythrocytes aging (Carelli-Alinovi et al., 1861; Dinarelli et al., 2018a,b, 2022) have combined morphological, ultrastructural, spectroscopic, and biochemical information and revealed important information on RBCs’ metabolism and on its interplay with their ability to process external stimuli throughout their aging cycle. These studies have evidenced different phenomena and adaptations occurring in healthy cells, compared to samples collected from patients with blood diseases, suggesting that these studies could be very useful in the characterization and early identification of these illnesses.

Favism arises from a genetic defect of the G6PD enzyme and results in a severe reduction of the erythrocytes’ reducing power that impairs the cells’ ability to respond to oxidative stresses. After the exposure to fava beans, aspirin, or a few other chemicals whose metabolites cause a remarkable oxidant insult, the patients experience acute hemolytic anemia due to massive erythrocytes’ lysis occurring both intra and extra-vascularly (Albano et al., 1984; Fischer et al., 1985; McMillan et al., 2001; Arese et al., 2012; Luzzatto and Seneca, 2014). A significant part of the damaged RBCs, however, is removed from the blood circulation by macrophages as “aged” cells. To further enhance the interplay between aging and RBCs’ removal in favism patients, it was observed that *in vivo*, after the administration of an oxidative stimulus, the first cells to lyse are the more senescent, likely because they have the lowest reducing power, or enzymatic activity (Morelli et al., 1978).

We have recently shown (Dinarelli et al., 2023) that G6PD deficient cells have a peculiar behavior along the aging pathway compared to normal RBCs. Indeed, favism erythrocytes display an initial higher fragility and occurrence of structural/morphological alterations compared to healthy cells but, at medium and long aging times, they evidence a stronger resistance to external stresses and a general higher resilience to lysis and aging. Remarkably, these significant behavioral differences can be associated to different regulations of the energy metabolism in the pathological cell, but this peculiar behavior cannot be observed nor characterized in detail without a cell aging study (Tzounakas et al., 2016; Reisz et al., 2017).

A more in-depth investigation of the erythrocytes’ aging would require the monitoring of the metabolic activity of the cells and the direct evaluation of their response to external insults such as ATP deprivation and aging. To perform such task, we employed a nanomotion sensor, which can monitor the evolution of the activity of single cells in real-time (Aghayee et al., 2013; Longo et al., 2013). This is a nanomechanical sensor which has already been used to study biological systems of diverse nature (Kasas et al., 2015a). For instance, the monitoring of the metabolic activity of bacteria has allowed performing rapidly and with high-accuracy the determination of their antibiotic susceptibility (Dinarelli et al., 2017; Stupar et al., 2017; Kasas et al., 2021), even for slowly-growing species (Mustazzolu et al., 2019) or of their virulence (Villalba et al., 2021). The nanomotion sensor has also been used to study the response of mammalian cells, representing a very interesting tools to study neurodegenerative diseases (Ruggeri et al., 2017; Lupoli et al., 2018) or the behavior of cancer cells (Kasas et al., 2015b; Wu et al., 2016), and with the promise to allow evaluating collective movements in small cell groups (Longo et al., 2021).

Due to the unique capabilities of this technique, in this work we have used the nanomotion sensor combined with conventional biochemical analyses, to evaluate better how the aging of healthy and favism erythrocytes influences their ability to respond to an external stimulus capable of activating a specific metabolic pathway. This investigation tool translates the nanoscale movements of cells produced by their metabolic activity into a measurable signal, providing a high time-resolution insight in the metabolic changes in cells exposed to different conditions.

## 2. Materials and methods

### 2.1. Chemicals and reagents

All the chemicals, reagents and functionalizing agents were of analytical grade and were supplied by Sigma Aldrich (Saint Louis, Missouri, United States).

### 2.2. RBC preparation and treatments

Blood from voluntary healthy donors (blood groups 0- and A+) was withdrawn and immediately diluted in a buffer phosphate solution (potassium phosphate 4 mM, NaCl 140 mM, pH 7.4) containing EDTA (1 mM) as anticoagulant. After a first centrifuge at 3000 rpm for 10 min the plasma containing supernatant was collected and stored at 4°C. The pellet of RBC was then washed three times to ensure the complete removal of plasma and buffy coat containing leukocytes. No antibiotics treatments were provided to the cells as well as no glucose was added to the buffer solution. The purified RBC were then stored at 20°C with a 20% hematocrit up to the day of use. Prior to the tests on the nanomotion sensor, the RBCs were washed twice with the same buffer without anticoagulant in order to remove the EDTA from the solution, then a 50 μL droplet of sample (at different concentration of cells and plasma) was deposited onto the substrate on the bottom of a petri dish, the cells were leaved for at least 30 min then gently washed to remove the extra cells, as final step the petri dish was filled with buffer solution.

### 2.3. Nanomotion sensor preparation

For all experiments, we used a Flex-AFM microscope (Nanosurf, Zurich, CH) mounted on top of an IX 50 Olympus inverted microscope (Olympus, Tokio, JP) and bearing a Progres MFCool digital camera (Jenoptik, Germany) for optical imaging. The entire AFM combined with the optical microscope system was placed in an environmental control chamber (a biological incubator) that ensured maintaining an optimal 37°C temperature and 5% CO_2_ conditions throughout each experiment. All the optical images were collected using a standard 40x objective in the phase-contrast modality, collecting images of the cells on the sensor every 20 s. For all our experiments, we used commercial tipless AFM cantilevers (NP-O10 Bruker, United States) with nominal spring constant of 0.12 N/m.

To ensure the adhesion and spread of the cells on the cantilevers, the sensors were chemically functionalized. The analysis of viable erythrocytes in liquid environment is particularly challenging due to the nature of these cells. Indeed, they tend to avoid the adhesion to surfaces and thus their membrane reacts immediately to many of the typically used chemical protocols used to immobilize biological systems. Thus, to have the cells adhere to the sensor’s surface we have employed a protocol that involves a very weak interaction with the membrane, that is, prior to introduction into the analysis chamber, the sensors were exposed to 50 μg/mL fibronectin for 15 min. After this period, the sensors were thoroughly washed with ultrapure water and used for the nanomotion experiments.

**Figure 1:**
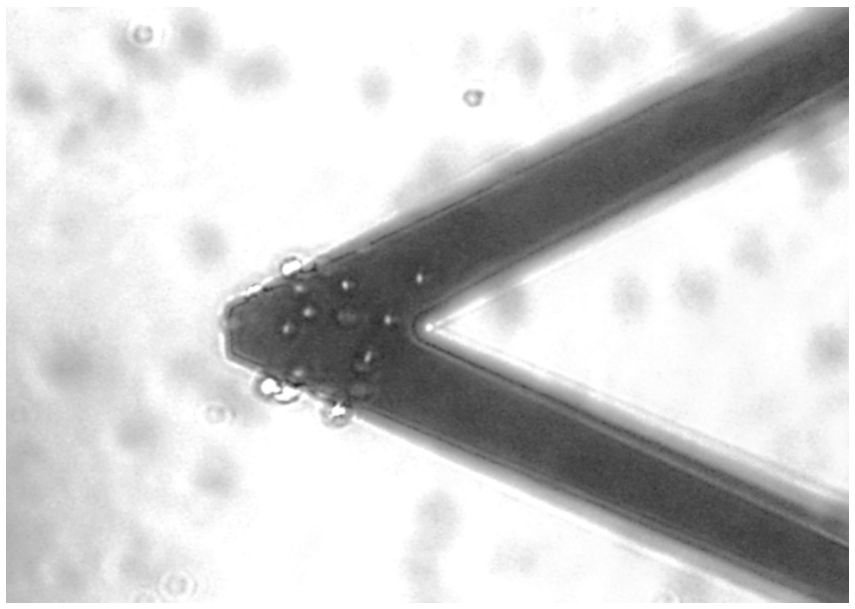
Optical image of a typical nanomotion sensor bearing approximately 20 single RBCs.

The RBCs were placed in a petri dish, immersed in a buffer without any Inosine-Pyruvate-Phosphate (IPP), which does not allow the cells to produce new ATP. In this environment, as soon as the cells had deposited on the bottom surface of the petri dish, we inserted the cantilever sensor. At this point, the cells were attached to the sensor using a “fishing” protocol typically employed in single cell force spectroscopy experiments and described in detail elsewhere (Kasas et al., 2015a). The AFM coarse motors allowed moving the cantilever over a cell and then the cantilever was brought in contact with the erythrocyte, through a controlled approach with a maximum applied force of 1 nN. This produces the attachment of the cell near sensor’s apical region. After 1 min, the cantilever was retracted, bearing on its surface the chosen cell. Typically, we chose to attach 10 to 25 single cells to the sensor, ensuring that all cells stayed on the apical region of the cantilever (Figure 1).

The number of the attached cells and their positioning on the sensor’s surface have indeed a role in the resulting nanomotion signal amplitude. Thus, we chose to work with a similar number of cells attached all to the most apical area of the sensor. Due to the attachment methodology, not all experiments were performed with the very same number of attached specimens and we performed normalization procedures to compare different experiments, which were similar to those described in detail in Longo et al. (2013).

After the attachment phase, we gradually retracted the sensor further away from the surface, to avoid interference from the substrate.

The cells were left 1 h on the sensor to stabilize and, during this period, we continuously measured the fluctuations of the cantilever, at 20 kHz acquisition frequency, and collected optical images of the cantilever’s surface (acquiring an image every 20 s) to determine the baseline state of the cells before the exposure to IPP medium. After this stabilization period, we very gently replaced the medium with the IPP solution, using the optical images to verify that this procedure did not cause the removal of the cells from the sensor.

The exposure to the IPP allowed the cells to produce ATP (DeVenuto et al., 1974). We followed this metabolic response by simultaneously using the nanomotion analyses and conventional optical microscopy. Due to experimental constraints, each experiment lasted between 2 and 3 h.

Subsequently, after this phase, the IPP was removed by replacing the medium with the previous IPP-free buffer. Finally, the cells were exposed to ultrapure water, which is a hypotonic solution, that caused their swelling, their inactivation and, ultimately, their explosion. This left just empty membranes on the sensors, that do not produce any active oscillation, allowing to determine the zero-level vibrations.

### 2.4. Data analysis and statistical evaluation

The time-dependent fluctuations of the sensor, linked to the metabolic activity of the biological specimens, were recorded using a custom LabView software to control a NI USB-4431 card (National Instruments, United States). The data analysis was carried out using a custom LabView software (Venturelli et al., 2020). The cantilever fluctuations were recorded with a sampling rate of 20 kHz.

**Figure 2:**
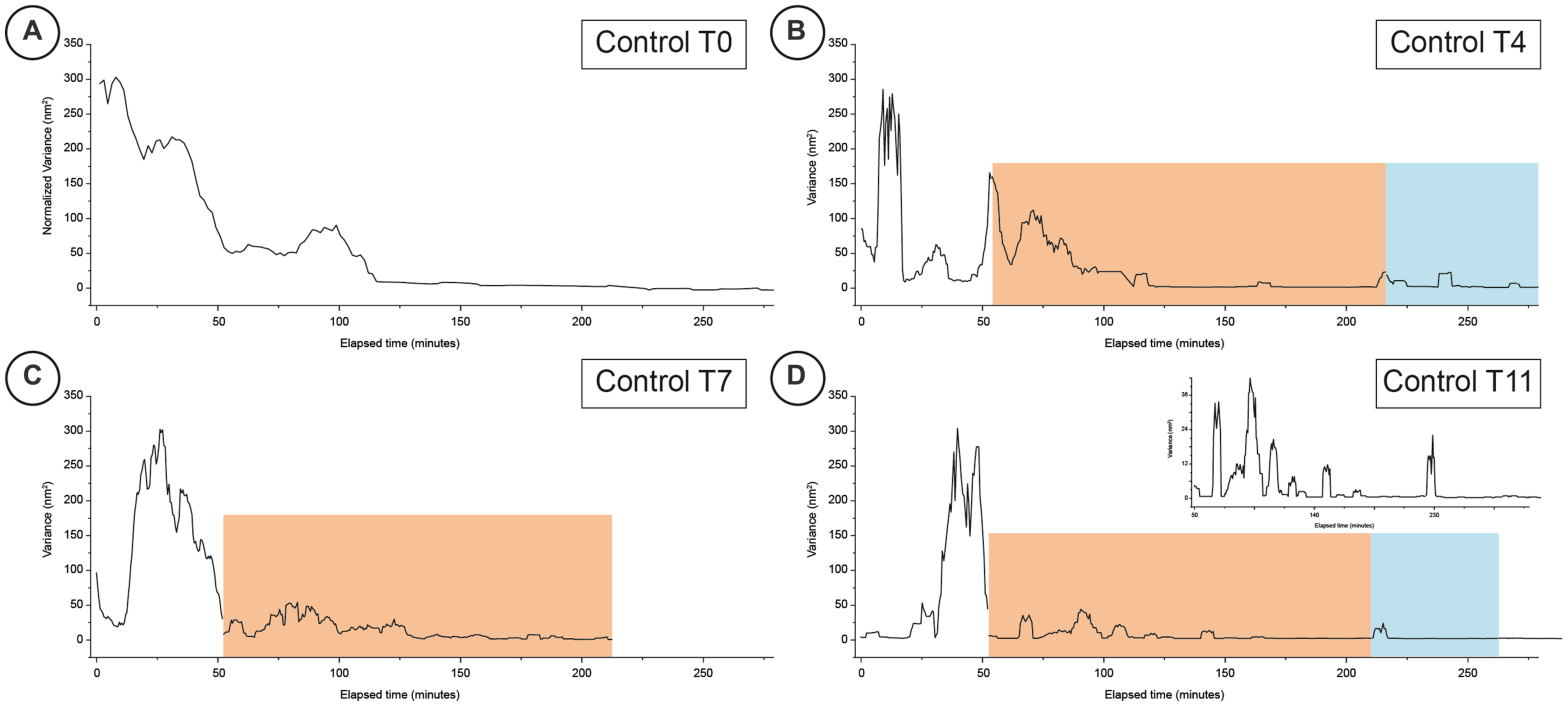
Typical time-resolved variance of nanomotion experiments performed on healthy cells at different time-points (Panels **A–D** for days 0, 4, 7 and 11 respectively). The orange areas indicate the response to the exposure to the IPP buffer while the blue areas depict the time-periods when the cells were subsequently exposed for a second time to the buffer with no IPP. The inset graph for panel **D** is a higher magnification of the fine structure of the corresponding nanomotion variance graph to highlight the finer variations of the data.

**Figure 3:**
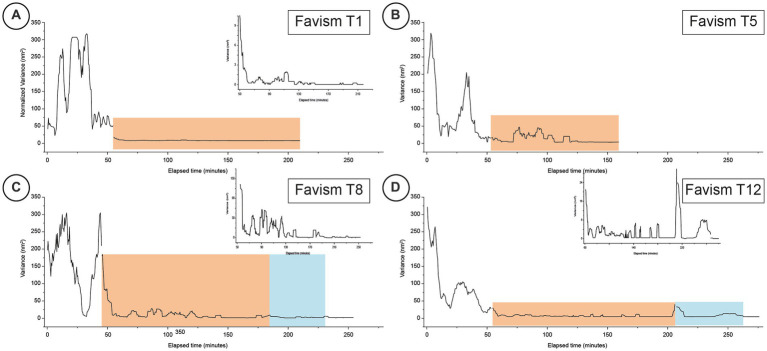
Typical time-resolved variance of nanomotion experiments performed on favism cells at different time-points (Panels **A–D** for days 1, 5, 8 and 12 respectively). The orange areas indicate the response to the exposure to the IPP buffer while the blue areas depict the time-periods when the cells were subsequently exposed for a second time to the buffer with no IPP. The inset graphs for panels **A,C,D** are higher magnifications of the fine structure of the corresponding nanomotion variance graphs to highlight the finer variations of the data.

The variance of the recorded fluctuations over time was calculated dividing the raw data in 35 s chunks. The graphs shown in Figures 2, 3 are obtained by performing a sweeping average of the variance values over 10 chunks.

All the nanomotion measurements were repeated minimally three times in independent experiments and the presented graphs represent the typical trends of the nanomotion variance over time.

**Figure 4:**
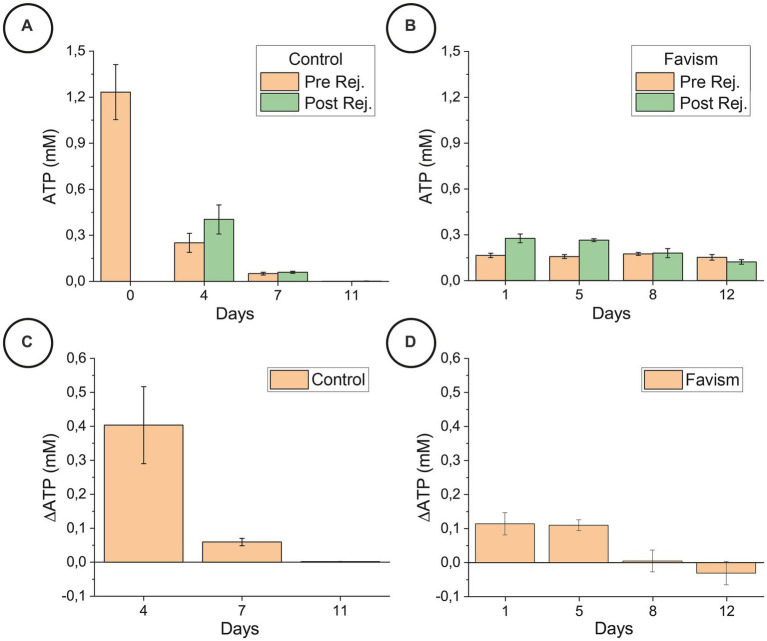
ATP content before and after the rejuvenation process, for the control (panel **A**) and favism (panel **B**) cells, and corresponding ATP variation (panels **C,D**) collected on the very same day of the correspondent nanomotion sensor experiments on a different aliquot of samples. The reported error is the standard deviation. The statistical significance of the differences was confirmed by ANOVA tests (*p* < 0.05).

The data reported in Figure 4 are ATP concentrations expressed in mM, normalized to a standard hematocrit of 45% and corrected according to the percentage of lysated cells (these operations were necessary in order to compare different experimental runs in a coherent way). The reported error is the standard deviation and the statistical significance was determined through one way ANOVA tests.

### 2.5. Rejuvenating solution preparation: IPP

The rejuvenating solution was prepared according to the method of DeVenuto et al. (1974) and was developed to provide the cells (the ones that are still capable to use the ATP synthetic pathway) with all the necessary components to synthesize ATP. The solution is called IPP (Inosine-Pyruvate-Phosphate) and contains 10 mM Inosine, 10 mM pyruvate, 75 mM sodium phosphate, 23 mM NaCl and 5 mM NaOH. According to our previous studies (Pompeo et al., 2010; Dinarelli et al., 2022) a complete recharging of ATP can be obtained after incubation in a solution containing buffer phosphate and IPP in ratio 1:5, for 3 h at 37°C.

### 2.6. ATP measurements

During the exposure to the nanomotion sensor, a vial containing RBCs at 20% of hematocrit, was incubated in the rejuvenation solution. After the 3 h of incubation the sample was centrifuged and washed twice in buffer phosphate to remove all the remaining IPP from the solution and then resuspended in phosphate buffer at 20% of hematocrit. A 200 μL aliquot of RBCs is then diluted 1:1 in buffer phosphate to proceed with the extraction of the ATP according to the following protocol:

- Centrifugation (3,200 rpm, 12 min), which removes completely the supernatant, followed by the addition of 1:5 (v/v ratio) of perchloric acid 0.6 M and, finally, gentle mixing for 1 min.
- Centrifugation (8,000 rpm, 8 min), to remove all the denatured proteins.
- 80 μL of the supernatant pipetted and diluted 1:6 (v/v ratio) in potassium carbonate 2.5 M; this neutralization step was performed in ice bath and last for 1 h, after which, 240 μL of the supernatant was collected.
- Centrifugation (11,000 rpm, 11 min), to remove all the remaining debris from the solution; 200 μL of the supernatant was then collected and immediately stored at −20°C until the day of the measurement.

The ATP measurements were performed with a Wallac 1,420 Viktor3 V plate reader by using the kit “Cell Titer-Glo luminescent Cell Viability Assay” from Promega. Briefly, for each sample three different aliquots of 65 μL were measured in three different 96 multi-well plates by adding 1:1 the Cell-Titer Glo reagent and then stored in dark environment for at least 20 min. Three different reading for each plate were performed after 20, 30 and 40 min from the mixing, to achieve the stability of the luminescent signal and avoid mixing errors. The correlation between counts and ATP content was performed through a series of calibration standards that were freshly prepared for each day of measurement. As previously stated, to achieve a direct correlation among different experimental runs, the values were normalized to a standard hematocrit of 45%.

## 3. Results and discussion

The nanomotion sensor is an innovative and very promising technique to obtain valuable insights into the metabolic behavior of biosystems by analyzing their nanoscale movements. The base postulate is that living biological systems perform functions linked to the cellular motility and that the metabolic energy of these systems is, at least partially, spent to perform such movements. This means that the correlation between metabolic energy consumption and movement allows to use the cellular movements to evaluate the status of a cell.

In this framework, RBCs represent a particularly valuable system since their metabolism is simplified by the fact that these cells produce their energy exclusively through glycolysis. Thus, these cells allow studying the metabolic activity of living specimens and its consequence on other activities of the biosystem. The nanomotion signal originates from many different metabolically-related phenomena that are concurrently happening inside the cell and on the cell membrane. Among these, an important role is arguably played by membrane flickering. These kinds of non-equilibrium membrane fluctuations, which are influenced by the dynamics and phosphorylation of the membrane-skeleton network, are certainly part of the nanomotion-collected signal as it is, itself, related to the intracellular ATP and to the flux of metabolic energy (Puckeridge et al., 2014).

In the present work, our approach has involved the analysis of the RBCs’ aging process, with a special focus on the changes in the cellular behavior associated to the residual intracellular energy. In particular, in our experimental conditions, the cells have been incubated in the absence of glucose or other nutrients that, during their aging, causes a continuous decrease of the intracellular ATP concentration (Girasole et al., 2010; Pompeo et al., 2010).

A fundamental aspect of the study was the rejuvenation procedure, in which, by incubating the cells in the presence of the chemical building blocks required for the production of energy (IPP solution), we forced the activation of a particular metabolic pattern. Indeed, for starved cells with little residual energy, and as long as their synthetic apparatuses remain efficient, the presence of IPP will make ATP synthesis the dominant metabolic pathway and this permits to analyze its characteristics. In this way, the nanomotion sensor can effectively provide unambiguous information associated with the duration and the energy compartmentation during a specific metabolic pattern.

Specifically, our present study focused on the comparison between the behavior of cells from a healthy donor and RBCs from a patient suffering from favism, a pathology that essentially affects the cell production of NADPH and, consequently its response to oxidative stress, but which we have recently shown to be deeply associated also with a peculiar regulation of energy metabolism and ATP consumption during the aging (Dinarelli et al., 2023).

In the first control experiments, we monitored for more than 2 h the freshly prepared cells (T0) in a medium that did not contain nutrients nor IPP. This allowed to determine the typical nano-motile characteristics of unperturbed erythrocytes over time (Figure 2A) which is characterized by large, collective, broad peaks slowly decreasing over time and lasting for nearly 2 h.

In the subsequent experiments, reported in Figures 2, 3, we monitored the behavior of normal and pathological cells at different aging times.

As shown in Figure 3, at T1 (Figure 3A) the favism cells’ response to the exposure to IPP was very limited as the cells, after the exposure to the IPP rich environment, reduced their activity very rapidly to a factor of 200.

Quite different was the response of the healthy cells monitored at T4 (Figure 2B) and of the favism cells at T5 (Figure 3B): the response to the IPP stimulation, for both samples, was intense (in the control it was comparable to the values measured before the exposure to IPP) with a nanomotion signal that started immediately after the exposure and that lasted at least 10–15 min. After this oscillation period, the samples reduced their movement yet, while the healthy RBCs’ movements went rapidly to low amplitude values, the cells from favism evidenced some continued oscillations even after more than 1 h from the exposure (bumps with measured motion intensity up to 1/10 of the unexposed cases). This particular observation suggests that the response to IPP, as measured by nanomotion, is associated to the cell’s ATP requirement (i.e., demand of new synthesis).

At higher aging times, for measurements performed after 7 or 8 days, the duration of these bumps increased but their intensity decreased over time. In fact, after 7 days (T7 of the healthy cells in Figure 2C and T8 of the favism cells in Figure 3C), in both healthy and favism cases the bumps had an intensity more than 100 times smaller than the average values measured in IPP-poor buffer but lasted longer, for up to 2 h in the case of the favism cells. Again, even at these higher aging times the healthy samples showed a faster response to the IPP, while the favism erythrocytes appeared to respond with a delay (or a transient) of approximately 10–15 min from the exposure to the IPP-rich medium. Both in the controls and in the pathological samples this first response to the exposure to the IPP lasted for approximately 1 h and was followed, in the control samples, by very little further activity. On the other hand, the favism samples showed sustained movements even after the end of the first bump (insets in panels C and D in Figures 2, 3).

The results of the experiment repeated at very long aging times (i.e., after 11/12 days) are reported in Figures 2D, 3D and showed similar characteristics: in the controls the oscillation were larger, while in the favism cells the signal was smaller but lasted for longer times.

The cellular response after the subsequent removal of the IPP buffer is quite interesting. Both the healthy and the favism cells exhibit a fast reaction to the new environment, with a fast bump that lasted several minutes but with a very low intensity (1/10 of the average IPP bumps), which then returned to base levels. Remarkably, after this first post-IPP bump, the favism cells produced smaller but still measurable subsequent vibrations, suggesting that, even at higher time-points, their activity is still present.

**Figure 5:**
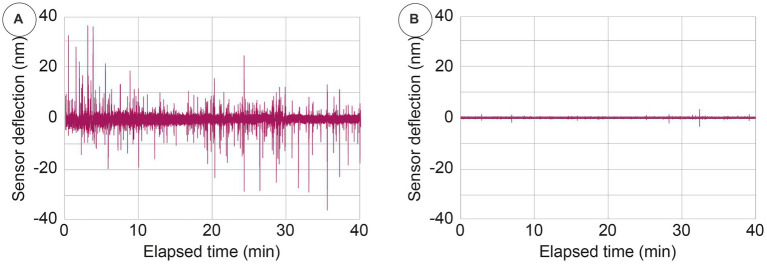
Typical nanomotion amplitude signal of viable (panel **A**) and exploded cells (panel **B**) obtained by exposing the cells to a hypotonic buffer.

To visualize the cellular activity in the nanomotion signal, we compared the nanomotion data of healthy cells with the very same cells after being exposed to a hypotonic buffer (namely ultrapure water), which has the effect of causing them to explode. The raw time-related fluctuations shown in Figure 5 evidence that all the fluctuations (ranging up to several tens of nanometers) that can be seen in presence of the viable cells have disappeared when these cells have been inactivated and have exploded. This depicts how the nanomotion variance originates from different and large life-correlated movements of the cells.

Simultaneously to the nanomotion sensor measurements, we collected a different aliquot of the same samples, and measured the intracellular ATP.

It is important to note that, while the rejuvenation for the ATP measurements was performed by maintaining the cells diluted 1:5 in IPP in a vial for 3 h, and the ATP content was measured after this incubation time, the nano-motile properties of the cells were measured on the nanomotion sensor during the exposure to the IPP. Consequently, the nanomotion and the ATP analyses are complementary to each other, with the first describing, in real time and nano-motile terms the dynamics associated to the metabolic production of ATP, while the second was used to define, in average over the whole sample, the effectiveness of the entire rejuvenation process.

Figure 4 shows that there is a large difference between the general ATP trend over time (Figures 4A,B) and the effectiveness of the rejuvenation procedure (Figures 4C,D) between the control and favism samples. The ATP trend over time is rapidly decreasing for the control cells while it remains almost constant (or slightly decreasing for longer aging times) in the favism erythrocytes. It is interesting to note that the starting content of ATP in the favism cells is very low with respect to the initial value of the healthy RBCs, and this is probably responsible for the higher fragility of pathological cells, but it is very well maintained over time. This is in good agreement with previously reported data on these cells (Dinarelli et al., 2023).

Another interesting behavior can be observed by comparing the effectiveness of the rejuvenation procedure in the two samples. Indeed, as shown in Figures 4C,D, the ATP reload is quite effective for the control cells in the first 5/6 days of aging (as also confirmed in our previous work (Dinarelli et al., 2018a)) while it becomes rather ineffective at longer aging times. On the other hand, the favism samples present a continuously decreasing amount of ATP generated and evidence that the effectiveness of the rejuvenation process is always lower, even at the beginning of the aging pathway, but the reload capability of these cells decreases less rapidly along the aging time. This substantial difference points to an innate resilience of the favism erythrocytes, which are known to have evidenced reduced lysis rates at higher aging times (Dinarelli et al., 2023).

All these considerations suggest that, for the favism erythrocytes, the amount of intracellular ATP is not as critical as for the control samples. Indeed, the pathological RBCs perform better with respect to the healthy ones at long aging times since they are capable to carry out their functions with ATP levels as low as 0.2 mM and which remain practically constant during the entire aging path. On the contrary, in the healthy cells the ATP decreased in a nearly exponential manner over time, and, at longer aging times, its amount has dropped to 0.001 mM.

The most interesting data here presented are clearly related to the behavior of the erythrocytes during the rejuvenation experiments. In the healthy RBCs, the cell movements associated with the ATP reload decrease at increasing aging, and in all cases the large movement phase occurs during, approximately, the first part of the IPP incubation. On the other hand, the behavior of the pathological cells shows movements of smaller amplitude, that are still present at longer times, both in terms of the IPP incubation phase and in terms of the increasing aging time. These data suggest that a different management of energy resources takes place in the favism samples and this idea is clearly supported by the measured ATP data. Indeed, the total amount of energy (ATP) present in favism cells, and consequently the part that the cells can spend for their nanoscale motion activity, is lower than in the controls.

In this sense, it seems that the amplitude of the nanomotion signals is correlated with the total energy of the cells which, evidently, decreases at increasing aging in all samples. Yet, in the specific case of the rejuvenation in IPP, it can be very useful to think this in terms of variation of ATP induced by the incubation (see Figures 4C,D) as this allows to highlight an interesting analogy between the reload efficiency and the oscillation curves. Indeed, the reload efficiency reported in Figures 4C,D indicate that for the control cells, we should have the largest oscillations at T4; that the nanomotion behavior of the T7 controls and of the T5 favism cells should be similar; and that the signal from the T11 control should be similar to that from favism at T8 and T12, with an even less intense oscillation.

In addition to this, by comparing controls and pathological erythrocytes we can highlight other behavioral differences as the aging time increases. Indeed, while in the control samples the strength of the nanoscale movement simply decreases following the trend of energy reduction, a sort of resilience of the nanomotion emerges in the favism erythrocytes and produces a persistence of the oscillations over the incubation time. This data indicates a greater behavioral variability in pathological erythrocytes which, especially as aging increases, consume less ATP (slower reduction of nanomotion amplitude) and are also able to manage the residual energy in a different way, for instance by continuing to produce nanoscale motion that is associated with energy consumption, even at very long aging times.

The characteristics of amplitude and duration of the nanomotion spots observed during the IPP incubation, especially at long aging times, suggest short phases of compulsive activation of the cellular motion, probably associated with specific and time-limited molecular or membrane dynamics events. In this sense, as aging increases (e.g., T8 and, above all, T12) the nanomotion signal of favism RBCs seems more characterized by sporadic and (likely) single cell activation events rather than by collective behaviors (see insets in Figure 3). In this sense, the behavior of favism RBCs could be associated with characteristics of resilience taking place in just some of the cells loaded on the cantilever. Interestingly, the results of a previous work, suggested that favism RBCs have a greater dispersion in terms of subpopulations compared to controls, and this effect determines a larger spread the cell properties, especially at the long aging times (Dinarelli et al., 2023).

One point that remains to be clarified, as the current data do not allow definitive conclusions, is whether the increase in cell nanoscale motion should be associated to the actual reload phase or, likely, to the cellular response following the synthesis of new ATP itself. In this sense, in future experiments, more refined measurements on the dynamics of ATP replenishment should be carried out, by measuring the ATP content at intermediate times of the incubation process. However, at least in the case of the IPP incubation, it is interesting to stress the association between the ATP recharge and the amount of energy used (or transferred) in nanomotion signals during the IPP incubation itself. This data, indeed, seems to suggest that is the energy surplus the parameter that is related to the nanomotion signal.

## 4. Conclusion

In this work we have employed for the first time a real-time nanomotion sensor to study RBCs and the activation of their ATP-producing metabolic pathways. These time-resolved analyses allowed evidencing the timing of the response over the aging pathway and the differences produced by favism in the cellular reactivity and resilience. In general terms, the nanomotion and ATP data indicate how the RBCs from favism patient exhibit a response to the rejuvenation which is less intense in terms of the amplitude of the oscillations but longer and sustained, suggesting a greater resistance to the aging-related insults. Indeed, at longer aging times, the movements of the pathological cells and their response to the rejuvenation procedure appears to be more persistent, when compared to the control erythrocytes.

Indeed, the nature of the RBCs ensure that the signal we have collected does not arise from filopodia and minimize the effects due to microscopical movements of the cells, while it suggests that the signal is a representation of smaller nanoscopic fluctuations of the membranes (e.g., flickering dynamics), driven directly from the ATP metabolism and associated to the functional activity of the specimens.

Since the rejuvenation procedure has the underlying effect of selecting the metabolically active cells within the population, our results confirm the recent observation that favism erythrocytes, at long aging times, evidence a stronger resistance to external stresses and a general higher resilience against the effects of the aging when compared to the healthy ones.

The characteristics of the nanomotion signal detected in the favism erythrocytes at long aging times and at long IPP incubation times present many markers of single, sudden, events, probably associated to specific molecular mechanisms or membrane non equilibrium dynamics, rather than the characteristics of massive collective behavior. This data suggest that the extensive nanoscale motion observed in the favism RBCs could be the characteristic of selected subpopulations of particularly resilient cells that can be observed as consequence of the larger population dis-homogeneity already evidenced for senescent RBCs in this pathology.

## Data availability statement

The raw data supporting the conclusions of this article will be made available by the authors, without undue reservation.

## Author contributions

MG, SD, and GL conceived and carried out the nanomotion experiments. MG conceived the ATP analyses. SD performed the ATP analyses. GL performed the nanomotion data analyses. All authors contributed to the article and approved the submitted version.

## Conflict of interest

The authors declare that the research was conducted in the absence of any commercial or financial relationships that could be construed as a potential conflict of interest.

## Publisher’s note

All claims expressed in this article are solely those of the authors and do not necessarily represent those of their affiliated organizations, or those of the publisher, the editors and the reviewers. Any product that may be evaluated in this article, or claim that may be made by its manufacturer, is not guaranteed or endorsed by the publisher.
